# The impact of urine pH on lithogenic risk profile in children with urolithiasis

**DOI:** 10.1007/s00467-025-07044-1

**Published:** 2025-11-14

**Authors:** Joanna Bagińska-Chyży, Jan K. Kirejczyk, Tadeusz Porowski, Carsten Alexander Wagner, Agata Korzeniecka-Kozerska

**Affiliations:** 1 Department of Pediatrics and Nephrology, Medical University of Bialystok, Waszyngtona Street 17, 15-274 Białystok, Poland; 2 Faculty of Health Sciences, University of Lomza, 14 Akademicka Street, 18-400 Lomza, Poland; 3 Department of Physiology, University of Zurych, Winterthurerstrasse 190, CH-8057 Zurich, Switzerland

**Keywords:** Children, Urinary pH, Kidney stones, Urolithiasis, Promoters, Inhibitors

## Abstract

**Background:**

Urinary pH is known to influence the solubility and excretion of lithogenic substances, yet its relationship with other metabolic parameters in pediatric stone formers remains underexplored. This study investigated the association between urinary pH and lithogenic risk factors in children and adolescents with urolithiasis compared to healthy controls.

**Methods:**

A total of 400 pediatric patients (ages 3–18 years) with urinary stones and 372 age- and sex-matched healthy controls were included. All participants completed a 24-h urine collection for comprehensive metabolic analysis. Parameters assessed included urinary pH, BMI z-score, urine volume, osmolality, excreted creatinine, GFR, and urinary excretion of calcium, ionized calcium, oxalate, phosphate, magnesium, citrate, and uric acid. Two lithogenic risk indices were also evaluated: Bonn Risk Index (BRI) and Upper Metastable Limit osmolality (UMLOsm).

**Results:**

Stone-formers demonstrated significantly higher urine volume, oxalate, calcium, ionized calcium, uric acid, and BRI compared to controls. In contrast, controls exhibited higher levels of urinary citrate, osmolality, and UMLOsm. Lower urine pH was associated with higher BMI z-scores and reduced urine volume. Calcium excretion increased with urine pH up to 7 before declining, whereas citrate and magnesium excretion rose at pH levels between 6.75 and 7.0, indicating a potential protective effect.

**Conclusions:**

Our findings highlight the importance of individualized dietary and lifestyle guidance in pediatric stone formers, with a focus on maintaining a healthy BMI, adequate hydration, and urine pH between 6.75 and 7.0 to support protective factors like citrate and magnesium.

**Graphical abstract:**

**Figure:**
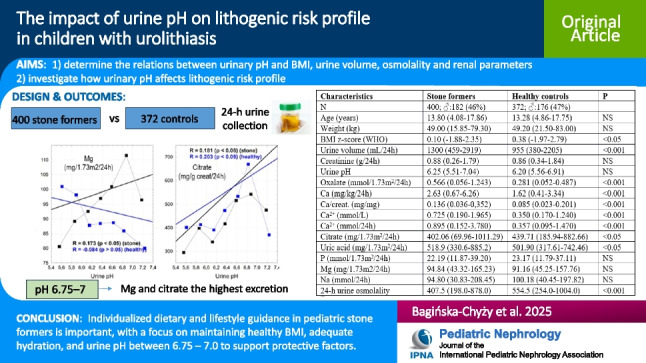
A higher resolution version of the Graphical abstract is available as  Supplementary information

**Supplementary Information:**

The online version contains supplementary material available at 10.1007/s00467-025-07044-1.

## Introduction

Urolithiasis is defined as the pathological formation of crystalline calculi within the urinary tract. Although historically regarded as a condition predominantly affecting the adult population, recent epidemiological trends indicate a rising incidence among pediatric patients [1]. In children, urolithiasis frequently exhibits a distinct pathophysiological profile when compared to adult cases. The underlying pathogenesis is multifactorial, involving a complex interplay of physicochemical, metabolic, and anatomical factors that lead to the formation of urinary calculi [2]. Stone formation typically begins with supersaturation of urine with lithogenic substances such as calcium, oxalate, uric acid, or cystine. This supersaturation promotes nucleation, where crystals form either spontaneously or on pre-existing surfaces such as epithelial cells or Randall’s plaques in the renal papillae [3]. Following nucleation, crystal growth and aggregation occur, facilitated by the absence or deficiency of natural urinary inhibitors of crystallization, such as citrate and magnesium. Additionally, alterations in urinary pH, volume, and flow dynamics influence stone formation by affecting solubility and crystal stability. Genetic predisposition and metabolic abnormalities, including hypercalciuria, hyperoxaluria, and hypocitraturia, further contribute to the risk.

Despite the expanding body of research suggesting that the incidence of urolithiasis in pediatric patients has increased over the past few decades, its underlying pathophysiology and risk factors remain insufficiently understood. One contributory factor that has been insufficiently investigated in the pediatric population is urinary pH.

Normal urine is slightly acidic, with a pH of approximately 6.0, though it can range from 4.5 to 8.0 depending on the time of day and dietary intake of food and water [4]. In children, urine tends to be less acidic than in adults, due to differences in diet [5]. During fasting or when consuming a high-protein diet, urine becomes more acidic. Conversely, alkaline urine is commonly excreted after meals rich in fruits, vegetables, and dairy products. Variations in urinary pH can also result from metabolic diseases or mutations in renal tubular transport pathways. For example, infants with distal renal tubular acidosis cannot acidify their urine, despite being systemically acidotic, and their urine pH consistently remains above 6.0. Distal renal tubular acidosis can arise from various causes and is often associated with hypercalciuria and kidney stone formation [6].

Changes in systemic acid–base homeostasis, such as chronic metabolic acidosis, as well as local renal pH, can alter ion channel function and influence the urinary concentrations of substances that promote (e.g., calcium and phosphate) or inhibit (e.g., citrate and magnesium) stone formation [7, 8]. In urolithiasis, urine pH is thought to affect various stages of stone formation, including crystallization, growth, and aggregation [9]. Additionally, urine pH influences the solubility of different metabolites and salts: acidic urine promotes the formation of uric acid or cystine stones, while alkaline urine increases the risk of calcium phosphate crystallization [10, 11]. In children with persistently alkaline urine, urinary tract infections caused by *Proteus* species, i.e. those that can split urea into ammonia and carbon dioxide, and thus alkalinize the urine, increase the risk of phosphate stone formation [12]. Urine pH should be measured immediately after collection or stored in a refrigerated state to prevent bacterial activity, which can alter the pH — typically increasing, though it may decrease in the presence of glucose [13]. As formation of renal stones is related to urine pH, its control is important in the management of urolithiasis.

The aims of this study were: first, to determine whether urinary pH correlates with patient BMI z-score, urine volume, and osmolality, excreted creatinine, and glomerular filtration rate (GFR); secondly, to investigate how urinary pH affects the lithogenic risk profile, and more specifically, excretion of calcium, ionized calcium, oxalate, phosphate, magnesium, citrate, uric acid, and how it affects two lithogenic risk indices, namely, the Bonn Risk Index (BRI) and Upper Metastable Limit osmolality (UMLOsm) in children and adolescents with urolithiasis and healthy controls.

## Methods

The study was conducted in the Department of Pediatrics and Nephrology, Children’s Clinical Hospital, Medical University of Bialystok, Poland. The study group consisted of 400 children and adolescents, aged 3–18 years (median 13.8 years), diagnosed with urinary stones of different compositions. All patients were recruited at their first presentation with urolithiasis, and a 24-h urine collection was obtained for initial metabolic evaluation. Children and adolescents who presented to the emergency department with symptoms of renal colic or urinary tract infection, and in whom urolithiasis was confirmed by imaging, underwent a 24-h urine metabolic evaluation one month after resolution of the acute episode and/or successful treatment of the infection. Children with nephrocalcinosis alone were not included. The presence of urinary tract stones was confirmed by imaging (ultrasound or CT) in all patients with urolithiasis. The exclusion criteria comprised abnormalities in dipstick urinalysis (Bayer Diagnostic, Bridgend, UK), any infections or medications, and inadequate 24-h urine collection assessed with urine creatinine excretion according to Remer et al. [14]. The control group comprised 372 age- and sex-matched healthy counterparts with neither kidney stones assessed by ultrasound nor laboratory urinary metabolic disturbances. In all participants, body weight and height were measured, and BMI z-scores were calculated. The participants provided a single 24-h urine collection at home while they were on their usual diet and carrying out their normal physical activities. After voiding, urine was stored in sterile, closed plastic receptacles at 4 °C to maintain original conditions without the addition of preservatives, and all measurements were conducted in a hospital laboratory within 4 h of the end of the collection period. After assessment of urine volume, the pH was measured using a laboratory CP-315 microcomputer pH-meter (Elmetron, Zabrze, Poland).

The groups of urolithiasis patients and controls were subsequently divided according to their urinary pH values into the following 7 subgroups: pH ≤ 5.75, 5.75 < pH ≤ 6.00, 6.00 < pH ≤ 6.25, 6.25 < pH ≤ 6.5, 6.50 < pH ≤ 6.75, 6.75 < pH ≤ 7.0 and pH > 7.00. Urine oxalate excretions were examined with an enzymatic–spectrophotometric method using a commercially available kit (Trinity Biotech, Berkeley Heights, NJ, USA). Urine calcium and creatinine were assessed using the Roche Cobas Integra 800 chemistry analyzer (Roche Diagnostics, Mannheim, Germany). The urinary concentration of Ca2 + was measured using Rapidlab 855 calcium ion-selective electrodes (Bayer, Leverkusen, Germany). Urine citrate levels were determined by an enzymatic method using a commercial set (Boehringer Mannheim/R-Biopharm, Darmstadt, Germany).

Two lithogenic risk indices were also evaluated: BRI and UMLOsm. BRI estimates the risk of spontaneous calcium oxalate crystallization in urine. BRI was determined according to the basic method of Laube et al. [15] using the modified analytical system described elsewhere [16]. After determination of Ca2+ using calcium ion-selective electrodes, the urine was titrated step by step with ammonium oxalate solution (Ox2−) in a computer-operated analytical system. When crystallized particles of CaOx caused a decrease in light transmission to 98% of the initial value, the computer application automatically stopped the titration process and calculated the value of BRI presented as [Ca2+] mmol/L/(Ox2−) mmol = 1/L.

After measurement of initial osmolality of urine (IUOsm) with a freezing point osmometer (model OS 3000; Marcel S.A., Poland), the urine samples were subjected to volume reduction in a vacuum rotavapor continued to the onset of an induced urinary crystallization. UMLOsm estimates the individual lithogenic capability and identifies people at risk of stone formation when exposed to dehydration. This indicator for a urine sample was assessed by its evaporation under strictly defined conditions until the onset of crystallization, causing an increase in urine turbidity. The UMLOsm of each urine sample was calculated based on its initial osmolality value (IUOsm) and the amount of water reduction. The lower the UMLOsm values, the higher the risk of crystallization and stone formation [17].

The protocol of the study was approved by the ethics committee of the Medical University of Bialystok (approval number: R-I-002/321/2016). Informed consent was obtained from the parents of all participants and from adolescents older than 16 years of age. Statistical analyses were performed using Statistica®, ver. 13.3 (StatSoft Inc, Tulsa, OK). The Mann–Whitney *U* test was used for comparisons between two independent parameters, and the correlations were made with the Spearman test. *P* values < 0.05 were considered to be statistically significant. *R* values of correlation coefficients ≤ 0.35 were considered to represent a weak relationship, 0.36–0.67 moderate association, and 0.68–1.0 strong correlation [18].

## Results

The comparisons of demographic, clinical, and urinary metabolic data between the urolithiasis and reference groups are summarized in Table 1. There were no statistically significant differences observed between genders. The median age and weight, urinary creatinine, pH, phosphate, magnesium, sodium, and osmolar load did not differ between groups. The urolithiasis group showed significantly higher urine volume, oxalate, calcium, calcium/creatinine ratio, ionized calcium, uric acid, and BRI than the reference group. The controls presented with higher urinary citrate, citrate/creatinine ratio, osmolality, and UMLOsm when compared to the patients.

**Table 1:** Summary of the comparisons of demographic, clinical and urinary metabolic data between the urolithiasis and reference group

Characteristics	Stone formers	Healthy controls	*P*
N	400; ♂:182 (46%)	372; ♂:176 (47%)	
Age (years)	13.80 (4.08–17.86)	13.28 (4.86–17.75)	NS
Weight (kg)	49.00 (15.85–79.30)	49.20 (21.50–83.00)	NS
BMI z-score (WHO)	0.10 (−1.88–2.35)	0.38 (−1.97–2.79)	< 0.05
Urine volume (mL/24 h)	1300 (459–2919)	955 (380–2205)	< 0.001
Urine volume (mL/kg/24 h)	29.43 (11.25–70.00)	21.51 (8.05–48.44)	< 0.001
Creatinine (g/24 h)	0.88 (0.26–1.79)	0.86 (0.34–1.84)	NS
Urine pH	6.25 (5.51–7.04)	6.20 (5.56–6.91)	NS
Ox (mmol/1.73 m^2^/24 h)	0.566 (0.056–1.243)	0.281 (0.052–0.487)	< 0.001
Ca (mg/kg/24 h)	2.63 (0.67–6.26)	1.62 (0.41–3.34)	< 0.001
Ca/creat. (mg/mg)	0.136 (0.036–0,352)	0.085 (0.023–0.201)	< 0.001
Ca^2+^ (mmol/L)	0.725 (0.190–1.965)	0.350 (0.170–1.240)	< 0.001
Ca^2+^ (mmol/24 h)	0.895 (0.152–3.780)	0.357 (0.095–1.470)	< 0.001
Citrate (mg/1.73 m^2^/24 h)	402.06 (69.96–1011.29)	439.71 (185.94–882.66)	< 0.05
Citrate/creat. (mg/g creat)	409.42 (63.65–1453.82)	448.83 (160.16–1098.71)	< 0.05
Uric acid (mg/1.73 m^2^/24 h)	518.9 (330.6–885.2)	501.90 (317.61–742.46)	< 0.05
P (mmol/1.73 m^2^/24 h)	22.19 (11.87–39.20)	23.17 (11.79–37.11)	NS
Mg (mg/1.73 m^2^/24 h)	94.84 (43.32–165.23)	91.16 (45.25–157.76)	NS
Na (mmol/24 h)	94.80 (30.83–208.45)	100.18 (40.45–197.82)	NS
BRI (1/L)	1.54 (0.10–8.69)	0.434 (0.068–4.125)	< 0.001
Osmolar load (mOsm/24 h)	529.6 (257.4–1141.4)	529.5 (233,9–958,8)	NS
24-h urine osmolality (IU_Osm_) (mOsm/kg H_2_O)	407.5 (198.0–878.0)	554.5 (254.0–1004.0)	< 0.001
UML_Osm_ (mOsm/kg H_2_O)	1928.0 (929.0–3584.0)	3031.4 (1994.3–5574.6)	< 0.001

Values are presented as median, with the range (5–95%) given in parenthesis

*BMI*, Body mass index; *Ox*, oxalate; *Ca*, calcium; *Ca*^*2*+^, ionized calcium; *P*, phosphate; *Mg*, magnesium; *Na*, natrium; *BRI*, Bonn Risk Index; *UML*_*Osm*_, Upper metastable limit osmolality; *NS*, not significant

**Fig. 1:**
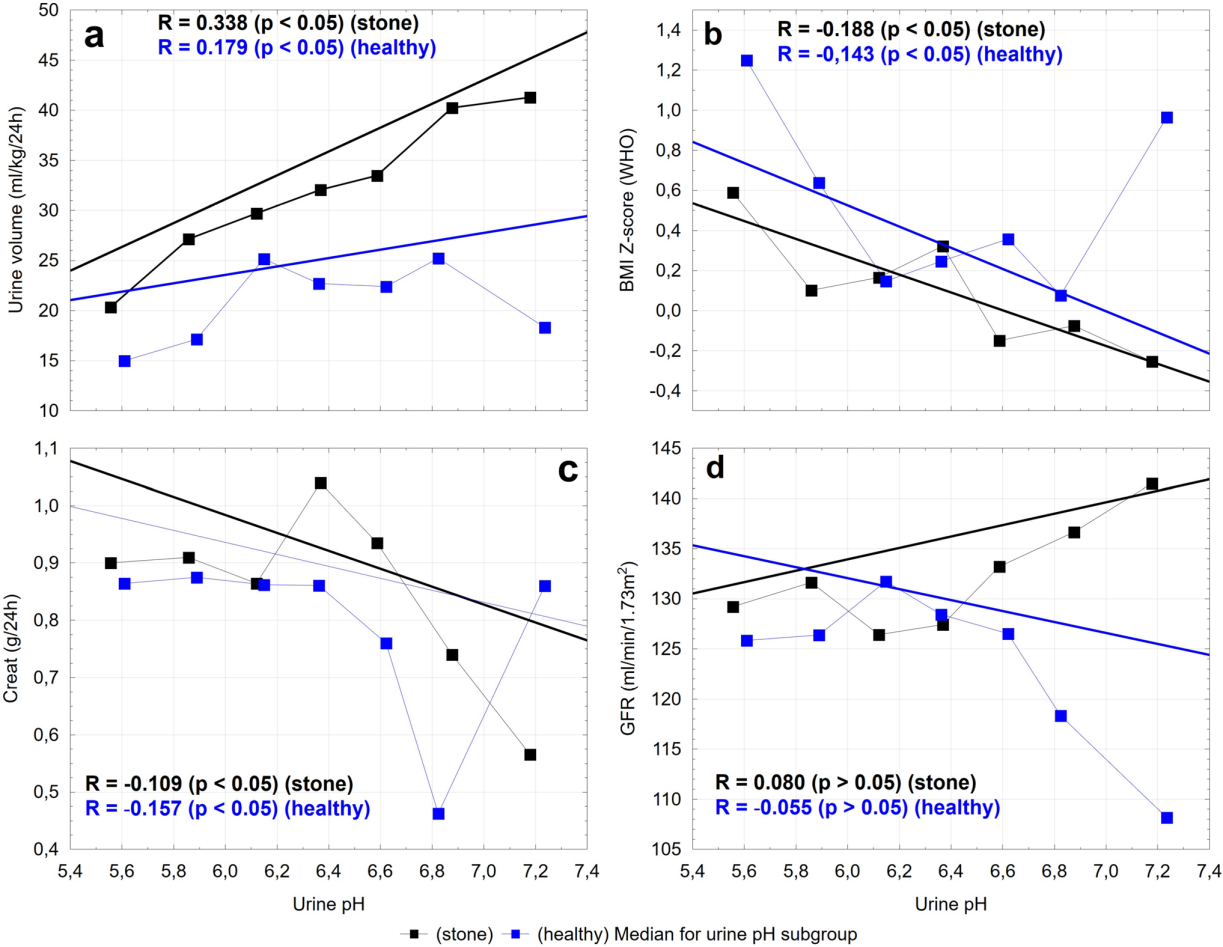
The urinary pH plotted against urine volume (**a**), BMI z-score (**b**), excreted creatinine (**c**), and GFR (**d**). The black and blue squares indicate the median values for each urine pH subgroup. A regression line, Spearman’s rank correlation coefficients and p values are shown for study and reference groups

**Fig. 2:**
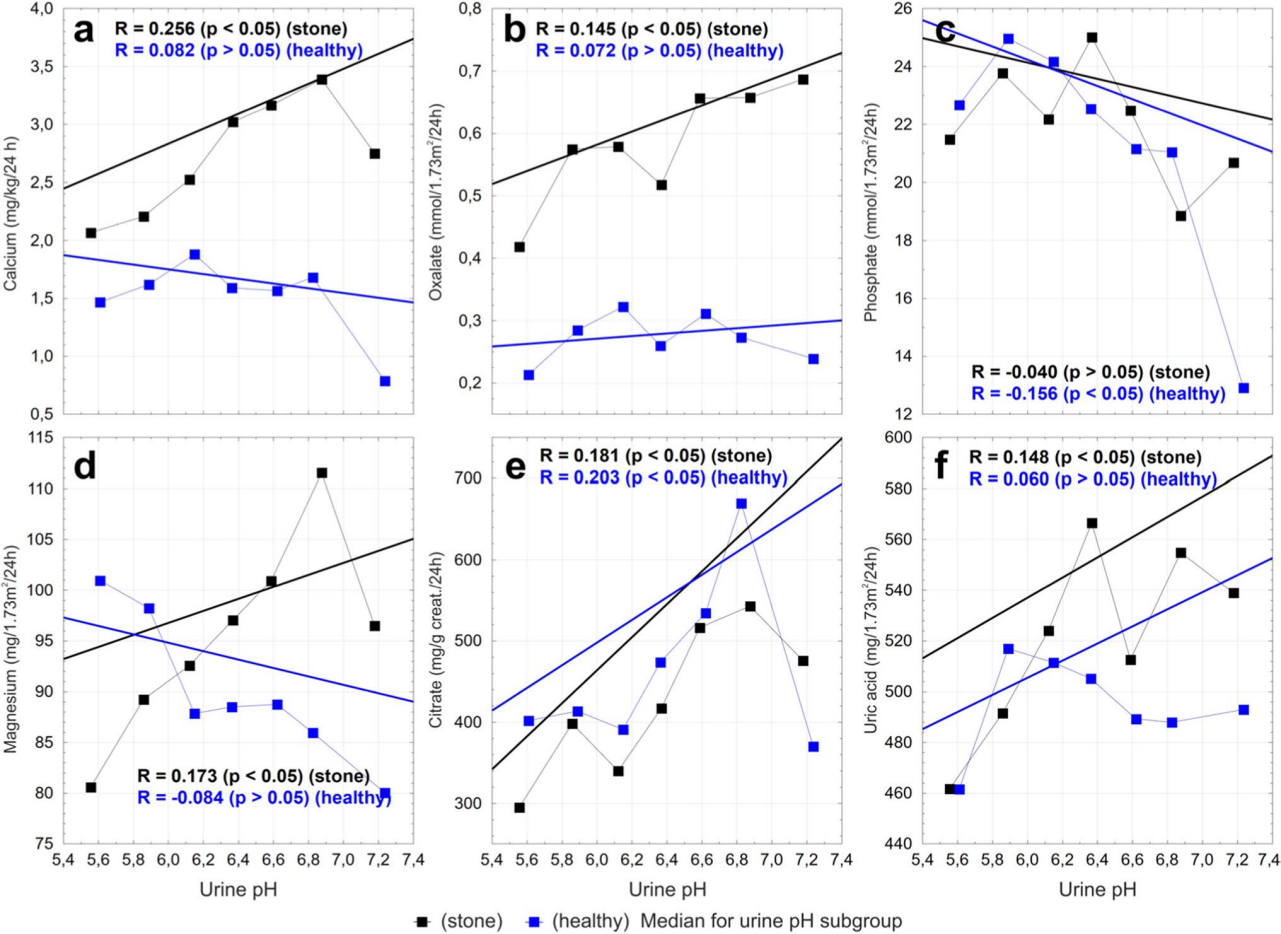
The urinary pH plotted against excreted calcium (**a**), oxalate (**b**), phosphate (**c**), magnesium (**d**), citrate (**e**), and uric acid (**f**). The black and blue squares indicate the median values for each urine pH subgroup. A regression line, Spearman’s rank correlation coefficients and *p* values are shown for study and reference groups

**Fig. 3:**
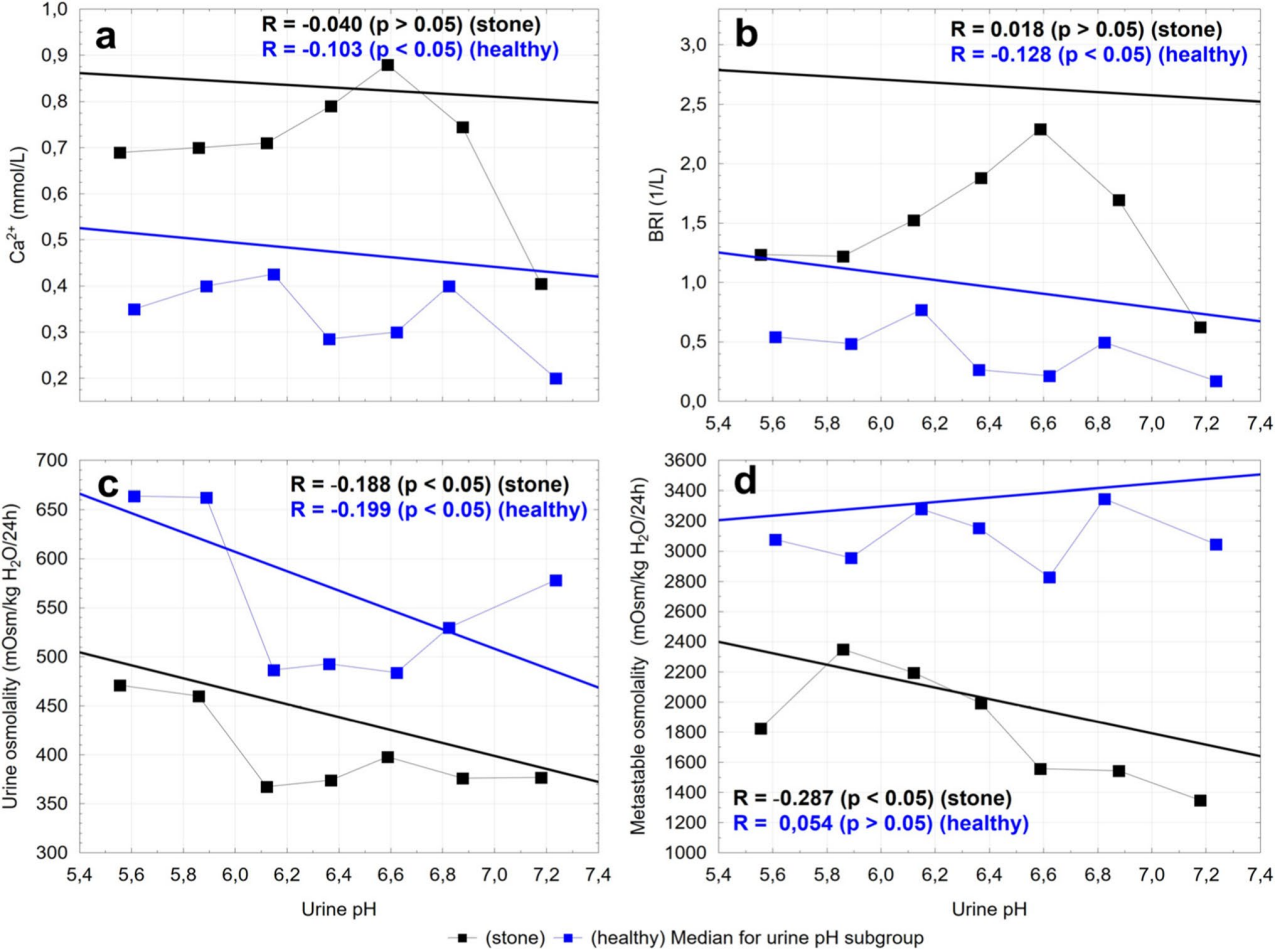
The urinary pH plotted against urinary ionized calcium (**a**), BRI (**b**), urine osmolality (**c**), and UML_Osm_ (**d**). The black and blue squares indicate the median values for each urine pH subgroup. A regression line, Spearman’s rank correlation coefficients and *p* values are shown for study and reference groups

The distribution of participants into subgroups according to urine pH in the study cohort and controls was as follows: pH ≤ 5.75 (51 and 49, respectively), 5.75 < pH ≤ 6.00 (67 and 74), 6.00 < pH ≤ 6.25 (78 and 84), 6.25 < pH ≤ 6.5 (85 and 76), 6.50 < pH ≤ 6.75 (69 and 55), 6.75 < pH ≤ 7.0 (28 and 25), pH > 7.00 (22 and 9, respectively). Across these subgroups, urinary pH demonstrated significant correlations with clinical, anthropometric, and biochemical parameters. These associations are illustrated in Figs. 1, 2, 3.

## Discussion

In the current study, we analyzed a large pediatric population to focus on the impact of urinary pH on the lithogenic risk profile. Maintaining a balanced urinary pH is critical for preventing crystallization, and interventions to acidify or alkalinize the urine are frequently utilized to manage or prevent stone recurrence. In the “Discussion” section, we systematically examine the relationship between the parameters studied and the risk of kidney stone formation in children.

### BMI

Although the connection between obesity and urolithiasis is well documented in adults, studies in the pediatric population are conflicting. Some authors have proposed that the increasing prevalence of childhood obesity may be contributing to the rise in pediatric urolithiasis [19, 20]. Like Fang et al. [19], our study found that the median body weight and BMI Z-score were within normal ranges for both the study and reference groups. This suggests that factors other than body weight may be associated with an increased risk of urolithiasis in children.

The observed negative correlations between BMI Z-score and urine pH in both stone formers and healthy controls align well with prior studies that have established obesity as a significant risk factor for nephrolithiasis. Higher BMI is associated with insulin resistance, which reduces renal ammonium excretion, thereby acidifying the urine [21]. This acidic urine environment promotes the crystallization of uric acid and other lithogenic solutes, contributing to the pathogenesis of kidney stones. Our findings, therefore, support the link between increased BMI and lower urine pH. Maintaining a normal BMI is important for all children, but it is especially crucial for those with urolithiasis.

### Urine volume

The median values of 24-h total urine volume as well as urine volume calculated per body weight were significantly higher in the patients when compared to the reference group. Similar findings have been reported by other researchers [22, 23], who also observed higher urine output in children with urolithiasis compared to healthy controls. One possible explanation for this increased urine output is elevated urinary calcium excretion. More calcium is filtered and excreted in the urine due to abnormal calcium-sensing receptor activity in the kidneys. This increased calcium load in the tubular lumen results in less effective reabsorption of sodium and water, which directly leads to a diuretic effect [24]. In the present study, stone formers had higher urine excretion of calcium, calcium/creatinine ratio, and ionized calcium. There is the possibility that intrinsic protective mechanisms, such as increased thirst leading to higher urine volume, might help mitigate stone formation. The second explanation may be connected with recommendations for urolithiasis patients about good hydration. Although all patients in this study had experienced a first stone episode, it remains possible that some had already received recommendations from other healthcare providers to increase their daily fluid intake as a preventive measure.

In our study, we observed a statistically significant positive correlation between urinary volume and urine pH. That means that lower urine volume is associated with more acidic urine, both of which are recognized as risk factors for stone formation.

### Urinary creatinine and GFR

Urinary creatinine is excreted by the kidneys at a relatively steady rate, making it a valuable biomarker for assessing and predicting renal function. In the context of kidney stones, several studies have noted higher urinary creatinine levels in stone formers compared to the general population. For example, Shen et al. [25] analyzed data from NHANES and found increased urinary creatinine levels in the kidney stone group. They suggested that higher urinary creatinine levels may indicate conditions conducive to stone formation by promoting urinary supersaturation, which may lead to crystallization in the renal pelvis. In our pediatric study, we did not observe elevated urinary excretion of creatinine in children with kidney stones. This difference may be attributed to factors such as diet and muscle mass, which influence creatinine production and make this cohort fundamentally different from adults. Importantly, as there was no difference in urinary creatinine levels, it reinforces its utility as a reliable denominator for calculating ratios of electrolyte excretion in 24-h urine collections.

The presence of kidney stones can sometimes cause urinary obstruction, leading to a decline in renal function and, in severe cases, irreversible kidney damage. When kidney function decreases significantly, urinary creatinine excretion may decline. As mentioned, in our investigation, no significant difference in creatinine excretion was observed between the two studied groups. The duration of the condition may play a pivotal role in the development of long-term complications associated with nephrolithiasis. Therefore, although urinary creatinine itself might not directly predict stone formation, it remains an essential marker for normalizing electrolyte excretion and evaluating overall kidney function, particularly in the context of obstructive nephropathy.

Moreover, analysis of the relationship between urinary pH and creatinine excretion demonstrated that both stone-forming patients and healthy controls with acidic urine exhibited slightly higher levels of urinary creatinine excretion compared to those with alkaline urine.

### Electrolytes excretion

Stone formers’ urinary excretions of calcium, oxalates, and uric acid were higher, whereas the excretion of citrate was lower in comparison to the reference group. The excretion of phosphorus, sodium, and magnesium did not differ between the studied groups.

#### Calcium and oxalate

Out of the observed 400 patients, calciuria within the normal interval was observed in the majority of cases. Only in 40% of cases did the urinary calcium excretion reach the level for hypercalciuria defined as greater than 4 mg/kg/24h. Even though calcium is a predominant crystalline constituent of kidney stones in children, it is necessary to emphasize that nephrolithiasis occurs both in patients with hypercalciuria and those with normocalciuria [26]. In our cohort, we measured the urinary excretion of both total calcium and its physiologically active form, ionized calcium. Both parameters were significantly higher in stone-forming individuals compared to the control group. Out of the observed 400 children, hyperoxaluria was observed in 48% of cases, defined as greater than 0.5 mmol/d/1.73 m^2^.

Calciuria can be influenced by urinary pH. Acidic urine (pH < 5.5) tends to promote the formation of calcium oxalate stones, whereas alkaline urine (pH > 7.5) may favor the formation of calcium phosphate stones. Although specific compositional data were unavailable for all participants, our prior analysis in the pediatric cohort indicated that calcium oxalate constituted the primary component in 73% of the stones [27]. Calcium oxalate stones are generally considered to be relatively independent of urinary pH, but pH extremes can still indirectly influence stone formation by affecting the solubility of other stone types or by altering calcium and oxalate handling [27]. In our study of stone-forming individuals, we observed a statistically significant positive correlation between urinary calcium and oxalate excretion with increasing urinary pH. Specifically, calcium excretion rose progressively with increasing urinary pH and peaked in a subgroup of 6.75 < pH ≤ 7.0, after which a marked decline was observed — suggesting a possible threshold effect. Ionized calcium levels remain relatively constant regardless of pH. Meanwhile, oxalate excretion continued to increase modestly across the pH range studied. Interestingly, in healthy controls, there was no significant correlation between urinary pH and the excretion of either calcium or oxalate. These findings support the hypothesis that in susceptible individuals, fine-tuning urinary pH — particularly maintaining it just above 7 — may help reduce the lithogenic risk by moderating calcium excretion.

#### Phosphorus

In stone formers, phosphate excretion remains relatively stable across different urinary pH levels. In healthy individuals, a more alkaline urinary environment was associated with lower phosphate excretion.

#### Uric acid

Although low urinary pH is classically associated with uric acid precipitation and stone formation, our study showed that stone-forming individuals exhibited higher urinary uric acid excretion even in the pH range 6.25–6.5, compared to healthy controls. This suggests that high uric acid excretion itself may be a risk factor independent of urine pH, particularly if supersaturation thresholds are reached. Therefore, in susceptible individuals, uric acid supersaturation can occur even at seemingly protective pH levels if excretion is sufficiently elevated.

#### Crystallization inhibitors: Magnesium and citrate

The magnesium excretion did not differ between studied groups. Urinary magnesium and pH are known to modulate urinary calcium excretion. In our study, both calcium and magnesium excretion increased with increasing urine pH. This supports the idea that urinary pH might influence calcium excretion directly or via its effect on magnesium [27, 28].

Stone-formers exhibited significantly lower citrate excretion compared to healthy controls. Citrate excretion tends to increase with higher urinary pH. This is consistent with known physiology — higher urinary pH promotes citrate excretion by reducing its tubular reabsorption. The highest concentration of crystallization inhibitors was present in the urinary pH range between 6.75–7.0.

#### Urine osmolality

Urine osmolality was significantly lower in stone formers in contrast to the control group. A negative correlation was observed between urine pH and urine osmolality in both groups. Thus, as urine pH decreases, urine osmolality tends to increase. This observation is consistent with prior studies demonstrating that acidic urine is typically more concentrated [29]. This relationship reflects normal renal physiology, wherein lower fluid intake or higher solute load leads to more acidic, concentrated urine. Conversely, alkaline urine is usually associated with higher fluid intake and a lower urine osmolality. Thus, our findings align well with existing literature and reinforce the importance of adequate hydration in stone prevention strategies.

When considering lithogenic risk indices, the urolithiasis group showed significantly higher BRI values than the control group, indicating a greater risk of calcium oxalate stone formation. Interestingly, the controls exhibited both higher urinary osmolality and higher UMLOsm compared to the patients, suggesting that higher urine concentration may not necessarily equate to a higher stone risk in healthy individuals. The greater osmolality in the control group is associated with lower urine volume compared to the stone-forming group, which in turn corresponds to lower calcium excretion. Despite the lower urine osmolality in stone-formers, crystallization occurred much earlier during sample evaporation, likely due to the high concentration of crystallization promoters and/or low levels of inhibitors. Additionally, a statistically significant negative correlation was noted between urine pH and UMLOsm in children with urolithiasis, suggesting that as urine pH increases, the risk of spontaneous crystallization decreases, although this effect may vary depending on the composition of the urinary calculi. Notably, the BRI appeared relatively constant across the urine pH range, with a slight peak around pH 6.6. The relationship and corresponding trends observed in the graphs for ionized calcium closely paralleled those of the BRI. Together, these results highlight how urine pH, osmolality, and lithogenic risk indices interact to influence stone risk, reinforcing hydration as a key preventive measure.

In summary, this study—the first to examine urine pH and lithogenic risk in a large pediatric cohort of stone formers—highlights several key risk factors for urolithiasis in children. We observed that higher BMI was significantly associated with lower urine pH, emphasizing the importance of maintaining a healthy BMI in children at risk for kidney stones. Additionally, lower urine volume correlated with decreased urine pH, both of which are well-known contributors to stone formation.

Stone-forming children exhibited higher excretion of calcium, oxalate, and uric acid, along with lower citrate excretion compared to controls. In contrast, phosphorus, sodium, and magnesium excretion did not differ significantly between groups. Notably, total calcium excretion rose progressively with increasing urinary pH and peaked in a subgroup of 6.75 < pH ≤ 7.0, after which a marked decline was observed, suggesting a threshold effect, while ionized calcium remained relatively stable across pH ranges. Oxalate excretion showed a modest increase across pH levels.

Importantly, both citrate and magnesium excretion increased at higher urine pH levels—particularly in the range of 6.75–7.0—highlighting a potential protective role against stone formation in this pH window. Furthermore, urine osmolality was lower in stone formers and negatively correlated with urine pH, reinforcing the importance of adequate hydration to maintain an alkaline, dilute urine that can help prevent stones.

This study has certain limitations. Systemic acid–base parameters such as blood pH and serum bicarbonate were not routinely assessed, which may have influenced urinary pH. Although each participant provided a single 24-h urine collection while on their usual diet and daily activities, dietary variations could still have affected urinary pH values. Despite these limitations, this study has important strengths. It represents the first large-scale investigation of urine pH and lithogenic risk factors in a pediatric stone-forming population. This study identifies several novel aspects of pediatric urolithiasis. A specific urine pH range of 6.75–7.0 was associated with lower calcium and higher citrate and magnesium excretion, suggesting a potential protective biochemical threshold for stone prevention in children. The nonlinear relationship between urinary calcium excretion and pH represents a new finding in pediatric populations. Additionally, the association of higher BMI and lower urine volume with lower urine pH highlights modifiable metabolic contributors to stone risk. By systematically examining metabolic parameters across narrow pH intervals, this study provides the first comprehensive analysis of pH-dependent urinary variation in pediatric stone formers.

## Conclusions

We believe that our findings underscore the importance of individualized dietary and lifestyle counselling in pediatric stone formers, focusing on healthy BMI maintenance, adequate fluid intake, and strategies to maintain urine pH between 6.75 and 7.0 to optimize the balance of crystallization inhibitors like citrate and magnesium. Monitoring urine osmolality and pH in clinical practice could serve as practical, non-invasive tools for assessing hydration status and guiding preventive strategies in at-risk children.

## Supplementary Information

Below is the link to the electronic supplementary material.

Graphical abstract (PPTX 286 KB)

## Data Availability

The datasets generated during and/or analysed during the current study are available from the corresponding author on reasonable request.
