# Supplementary material for: The impact of urine pH on lithogenic risk profile in children with urolithiasis
Source: Pediatr Nephrol. 2025 Nov 14;41(3):755–64. doi: 10.1007/s00467-025-07044-1 (PMC12852288; doi:10.1007/s00467-025-07044-1)
Supplement: Supplementary file 1 — Graphical abstract (PPTX 286 KB) [file 467_2025_7044_MOESM1_ESM.pptx]

## Slide 1
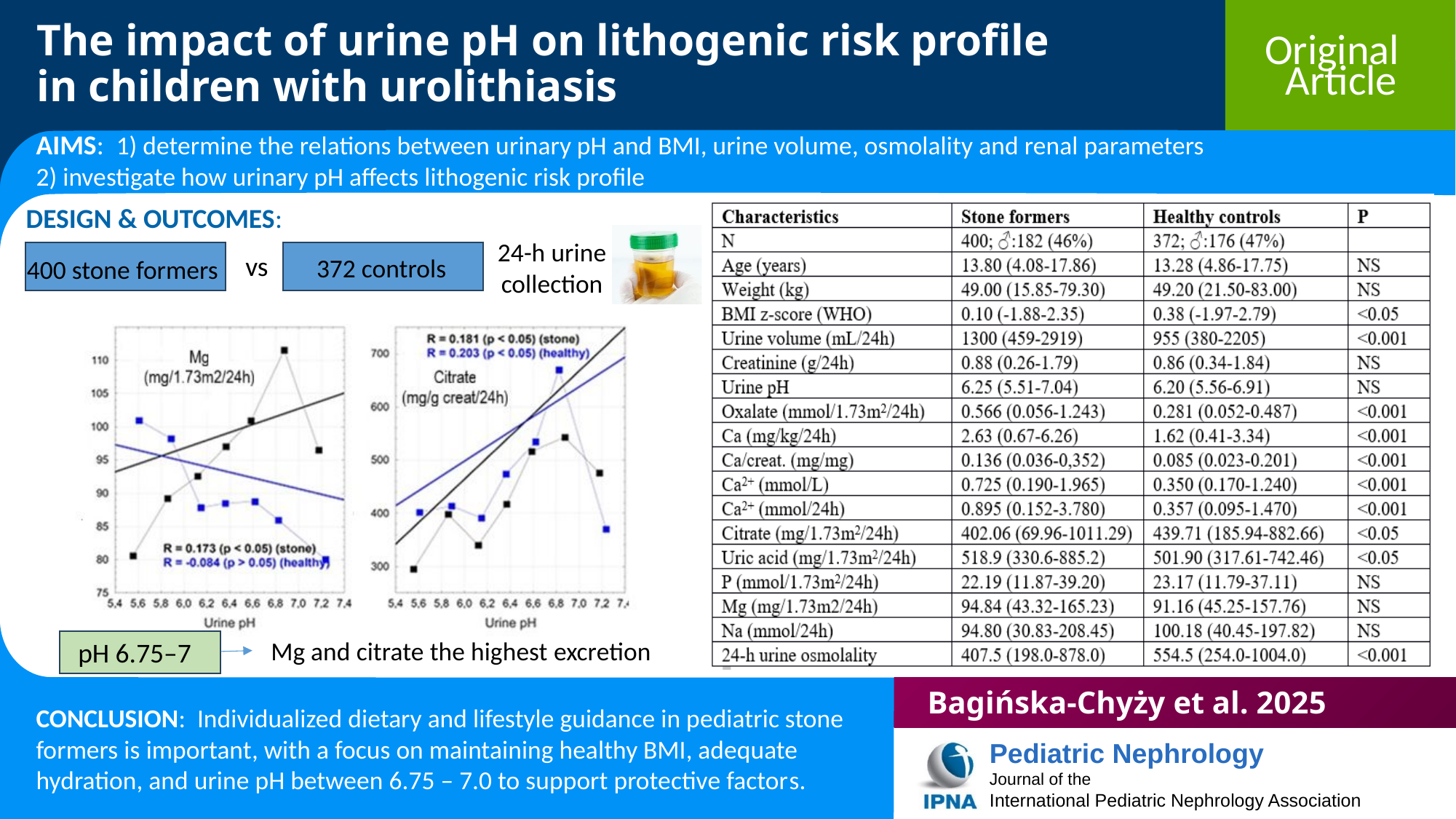

The impact of urine pH on lithogenic risk profile
in children with urolithiasis
AIMS: 1) determine the relations between urinary pH and BMI, urine volume, osmolality and renal parameters
2) investigate how urinary pH affects lithogenic risk profile
DESIGN & OUTCOMES:
24-h urine collection
372 controls
vs
400 stone formers
Mg and citrate the highest excretion
pH 6.75–7
Bagińska-Chyży et al. 2025
CONCLUSION: Individualized dietary and lifestyle guidance in pediatric stone formers is important, with a focus on maintaining healthy BMI, adequate hydration, and urine pH between 6.75 – 7.0 to support protective factors.
